# Relentless placoid chorioretinitis associated with Crohn's disease and secondary MEWDS: a case report

**DOI:** 10.1016/j.ajoc.2026.102550

**Published:** 2026-02-20

**Authors:** Marion Sagnard, Pierre Gascon, Alban Comet

**Affiliations:** Ophthalmology Department, Hopital Nord, Aix Marseille University, Marseille, France

**Keywords:** Relentless placoid chorioretinitis, Crohn's disease, Multiple evanescent white dot syndrome, Uveitis, Anti–TNF therapy, Multimodal imaging

## Abstract

**Purpose:**

To describe a rare case of relentless placoid chorioretinitis (RPC) associated with Crohn's disease, complicated by secondary multiple evanescent white dot syndrome (MEWDS).

**Observations:**

A 21-year-old man presented with acute, painless vision loss in the right eye. Multimodal retinal imaging showed numerous active and atrophic placoid lesions involving both the posterior pole and the retinal periphery, consistent with RPC. Systemic evaluation revealed Crohn's disease. Despite intravenous and oral corticosteroids, new extramacular lesions developed and fundus autofluorescence showed stippling compatible with secondary MEWDS. Introduction of anti–tumor necrosis factor (anti-TNF) therapy for Crohn's disease stabilized ocular inflammation. Visual prognosis remained poor in the affected eye and preserved in the fellow eye.

**Conclusions and Importance:**

This case highlights an association between RPC and Crohn's disease and supports an autoimmune mechanism. Early systemic evaluation and timely initiation of corticosteroid-sparing immunomodulatory therapy may help prevent recurrences and vision-threatening complications.

## 1 Introduction

Relentless placoid chorioretinitis is a rare, vision-threatening posterior uveitis defined by numerous scattered placoid chorioretinal lesions affecting both the posterior pole and the retinal periphery, evolving through different chronological stages with prolonged or recurrent activity.^1^ First described by Jones et al., in 2000,^2^ RPC is now considered distinct from acute posterior multifocal placoid pigment epitheliopathy (APMPPE) and serpiginous choroiditis (SC).^3^ Although the pathogenesis remains uncertain, autoimmune associations have been reported.^1^ We present a case of RPC associated with Crohn's disease, complicated by MEWDS, which underscores the potential link between ocular and systemic autoimmune disease.

## 2 Case description

**Fig. 1:**
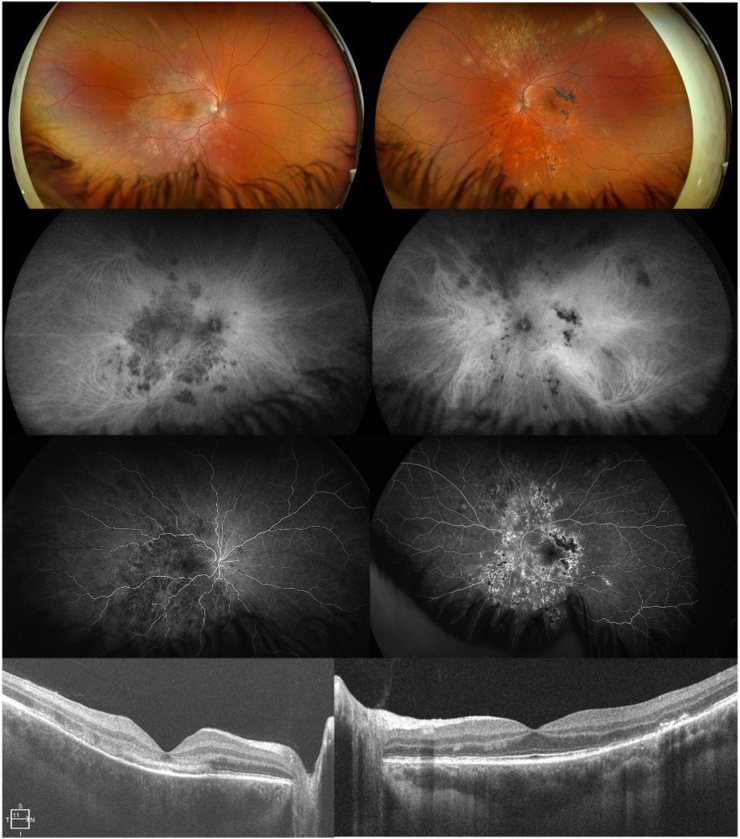
Multimodal imaging of both eyes of a young adult man with relentless placoid chorioretinitis in the early active stage. Widefield color fundus photography (upper panels) shows numerous confluent creamy placoid chorioretinal lesions at the posterior pole of the right eye, and multiple confluent, partially pigmented chorioretinal scars extending from the posterior pole to the periphery of the left eye, sparing the macula, along with creamy placoid lesions in the peripheral retina. Early-phase fluorescein and indocyanine green angiograms (middle panels) reveal multiple confluent hypofluorescent and hypocyanotic lesions on the right eye. Early-phase fluorescein angiography further reveals areas of hyperfluorescence corresponding to underlying atrophic changes in the left eye. Spectral-domain optical coherence tomography (lower panels) demonstrates a homogeneous band-shaped change in the superficial choroid consistent with inflammatory infiltration, as well as hyper-reflectivity with disruption of the outer retinal layers in the right eye. Multifocal thickening, irregularity, and even fibrous detachments of the retinal pigment epithelium are seen at the level of the chorioretinal scars, associated with multifocal disruptions and thinning of the outer retinal layers in the left eye. (For interpretation of the references to color in this figure legend, the reader is referred to the Web version of this article.)

A 21-year-old male presented with sudden, painless vision loss in the right eye for five days. Best corrected visual acuity was counting fingers at 1 m OD and 20/20 OS. Fundus examination revealed a large placoid lesion in the posterior pole OD and multiple active and atrophic lesions OS. Multimodal imaging demonstrated characteristic features of RPC: hypofluorescence with late staining on fluorescein angiography (FA), persistent hypocyanosis on indocyanine green angiography (ICGA), outer retinal disruption and ASHH (Angular Sign of Henle fiber layer Hyperreflectivity) on SD-OCT, and hyperautofluorescent halos on fundus autofluorescence (FAF) (Fig. 1).

**Fig. 2:**
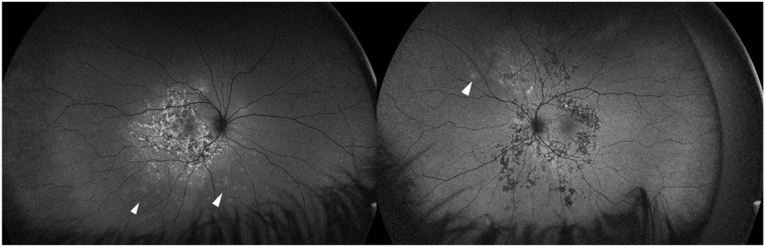
Fundus autofluorescence imaging shows diffuse hyperautofluorescence of early active placoid lesions and stippled hyperautofluorescence of older lesions with some lesions demonstrating central hypoautofluorescence and a surrounding rim of hyperautofluorescence. The white arrowheads show areas of hyperautofluorescence in the mid-periphery next to active lesion suggestive of secondary MEWDS.

Systemic evaluation identified chronic gastrointestinal symptoms, and colonoscopy with elevated fecal calprotectin confirmed Crohn's disease. Infectious causes such as syphilis and tuberculosis were excluded. The patient was treated with intravenous methylprednisolone (1 g/day for 3 days) followed by an oral prednisone taper. Despite therapy, new extramacular lesions developed bilaterally, and FAF revealed hyperautofluorescent spots consistent with secondary MEWDS (Fig. 2). Anti-TNF-α therapy was subsequently introduced, leading to disease stabilization.

Visual acuity remained poor in the right eye, while OS was preserved. Retinal imaging confirmed progressive atrophy and cicatricial changes consistent with RPC. Anti-TNF-α therapy, introduced for Crohn's disease, was associated with stabilization of ocular lesions and prevention of further recurrences.

## 3 Discussion

RPC is a distinct posterior uveitis entity predominantly affecting young adults (median age 23 years).^1^ Its defining features are multiple, widespread placoid lesions at different stages of activity, distinguishing it from APMPPE and SC^1^, ^2^, ^3^. Multimodal imaging is essential, with FA showing a 'block early, stain late' pattern, ICGA revealing persistent hypocyanosis, and OCT demonstrating outer retinal changes such as ASHH and BALAD. FAF may show cockade-like hyperautofluorescence.^1^

Systemic associations are uncommon but include thyroiditis, multiple sclerosis, and vasculitis.^4^ Our case is notable for the association with Crohn's disease, consistent with the autoimmune hypothesis underlying RPC pathogenesis. Therapeutically, corticosteroids alone are often insufficient, with most patients requiring early corticosteroid-sparing immunosuppressants.^1^ Biologic therapies, particularly adalimumab and infliximab, have shown efficacy in refractory cases. In this patient, anti-TNF-α therapy for Crohn's disease also achieved stabilization of RPC^1^.

## 4 Conclusion

RPC is a rare posterior uveitis that can cause significant visual morbidity. Its clinical course is often prolonged and relapsing, requiring aggressive immunosuppressive therapy. This case highlights an association between RPC and Crohn's disease, emphasizing the need for systemic evaluation in young patients presenting with RPC. Early initiation of immunomodulatory therapy is critical to prevent vision-threatening complications.

## CRediT authorship contribution statement

**Marion Sagnard:** Conceptualization, Data curation, Writing – original draft. **Pierre Gascon:** Supervision, Validation. **Alban Comet:** Data curation, Funding acquisition, Supervision, Validation.

## Patient consent

Written consent to publish this case has not been obtained. This report does not contain any personal identifying information.

## Conflicts of interest

The following authors have no financial disclosures: M.S., P.G., A.C.

## Authorship

All authors attest that they meet the current ICMJE criteria for Authorship.

## Funding

No funding or grant support.

## Declaration of competing interest

The authors declare that they have no known competing financial interests or personal relationships that could have appeared to influence the work reported in this paper.
